# Genetic parameters of functional longevity and associated traits in Italian Charolais and Limousine breeds

**DOI:** 10.1093/jas/skae354

**Published:** 2024-11-18

**Authors:** Simone Callegaro, Francesco Tiezzi, Christian Maltecca, Maria Chiara Fabbri, Riccardo Bozzi

**Affiliations:** Department of Agriculture, Food, Environment, and Forestry (DAGRI), University of Florence, Florence 50144, Italy; Department of Agriculture, Food, Environment, and Forestry (DAGRI), University of Florence, Florence 50144, Italy; Department of Agriculture, Food, Environment, and Forestry (DAGRI), University of Florence, Florence 50144, Italy; Department of Animal Science, North Carolina State University, Raleigh, NC 27695, USA; Department of Agriculture, Food, Environment, and Forestry (DAGRI), University of Florence, Florence 50144, Italy; Department of Agriculture, Food, Environment, and Forestry (DAGRI), University of Florence, Florence 50144, Italy

**Keywords:** beef cattle, conformation traits, functional longevity, genetic association, single step

## Abstract

This study aimed to estimate the genetic parameters of stayability (**STAY**) at different calvings using a single-step genomic best linear unbiased prediction (**ssGBLUP**) approach, comparing Gaussian-linear and threshold models in Italian Charolais and Limousine beef cattle. It also examined the genetic relationship between STAY and other traits to identify potential indicators of longevity and assessed the impact of STAY selection on economically important traits. STAY, a key trait for farm profitability, is defined as the probability of a cow surviving and remaining productive in the herd until a determined age. We evaluated STAY from the second to third calving and subsequent intervals (e.g., STAY23, STAY78), along with two fertility traits and several conformation traits. Data included 47,362 Limousine cows and 9,174 Charolais cows from 2,471 to 1,774 herds, respectively, born between 1977 and 2023. Analyses were performed fitting univariate threshold and Gaussian-linear animal models to estimate genetic parameters for STAY traits (STAY2 to STAY8) using ssGBLUP. Also, bivariate models were used to estimate genetic correlations between STAY and fertility and conformation traits. Heritabilities for STAY ranged from 0.13 to 0.11 and from 0.21 to 0.14 for Limousine, and from 0.14 to 0.11 and from 0.21 to 0.19 for Charolais, using Gaussian-linear and threshold models, respectively. Significant re-ranking of genotyped sires based on STAY traits was observed, particularly for more distant calvings (STAY8) compared to earlier ones (STAY3), indicating that STAY traits are genetically distinct. Genetic correlations were positive between STAY and conformation traits for Limousine. In Charolais, many traits were uncorrelated, but some conformation traits showed positive correlations, except for rump convexity, which had negative correlations with STAY. In conclusion, the heritability estimates of STAY suggests that genetic improvement for longevity in Limousine and Charolais herds is feasible. Selecting sires with consistently high genomic breeding values for STAY across early and late calvings highlights the importance of long-term longevity. Genetic correlations indicate that selection based on conformation traits could enhance herd survival by improving cow resilience for the Limousine. Instead for the Charolais some conformation traits showed positive correlations with STAY, while rump convexity had negative association, potentially affecting longevity.

## Teaser

Determine how genetic selection can improve the longevity and productivity of beef cattle herds. Our study explores the genetic background of functional longevity in Italian Charolais and Limousine breeds, aiming to reduce costs and enhance farm profitability through breeding strategies.

## Introduction

In the majority of beef cattle production systems, the duration of a cow’s productive life, the rate of calf production per cow over time, and the weight of calves at weaning are crucial economic aspects (Santana et al., 2013). A large part of the costs of beef cattle production are represented by the maintenance of heifers for replacement (Newman et al., 1992). Therefore, to reduce costs derived from replacement or culling, increasing herd longevity is one of the most effective strategies (Rizzo et al., 2015). A cattle farmer must ensure that replacements remain in the herd long enough to produce sufficient calves to offset the costs of their rearing and maintenance (Silva et al., 2024).

Stayability (**STAY**) is a crucial trait for on-farm profitability due to its association with specific costs and herd efficiency. Hudson and Van Vleck (1981) defined STAY as the likelihood of a cow surviving and producing in the herd until a specified age, provided that animals have the opportunity to reach that age. It serves as a measure of survival and is typically expressed as a binary trait (“yes” or “no”; “1” or “0”), indicating whether animals remain in the herd or not, without the need for recording culling dates. Predicting the genetic value of STAY offers an opportunity to reduce costs and increase farm income. Selecting for high genetic values of STAY can reduce turnover rates and decrease replacement costs of the farm. This could improve average herd efficiency, by extending the productive lifespan of cows. Integrating this trait into genetic evaluations enables the identification of parents whose daughters are more likely to remain productive for extended periods in the herd (Van Melis et al., 2007; Jamrozik et al., 2013).

Threshold models are specifically designed for binary traits like STAY, but they may have limitations in terms of complexity and interpretability compared to linear mixed models (Gianola, 1982). In contrast, linear mixed models provide an alternative for analyzing continuous and categorical traits, providing additional insights into genetic variation. By comparing these models is possible to determine which approach provides a more accurate representation of STAY traits across different calvings. This comparison can help to identify the most suitable method for genetic evaluations in beef cattle and enhance the accuracy of genetic evaluation for longevity.

The primary challenge in STAY genetic evaluations stems from the time required for a female to exhibit the phenotype at a certain age, potentially leading to lower accuracies in young bulls (Hudson and Van Vleck, 1981; Silva et al., 2018). Longevity selection is further complicated by the trait’s low heritability and the influence of environmental and management factors, along with genetics. Assessing traits like STAY within a multiple-trait model can reduce selection random errors and improve the accuracy of genetic parameter estimates for other traits (Pollak et al., 1984). Indeed, incorporating animals no longer in the herd into the analysis can reduce the number of records removed from the dataset (Pollak et al., 1984). Employing STAY within a multiple-trait approach could potentially enhance longevity in beef cattle. Furthermore, conformation traits may contribute to herd survival rates due to their correlation with longevity (Hu et al., 2021; Buonaiuto et al., 2023). These traits, which encompass aspects like body size, shape, and bone structure, contribute to individuals’ overall health and resilience, thereby impacting the survival rates of the entire herd. Moreover, fertility traits can enhance average herd longevity, due to a low to moderate genetic association with longevity (Hu et al., 2021, 2023). Fertility can be used as indirect indicators to select cows with greater potential for early longevity selection (Hu et al., 2021). A study found that older heifers had a higher culling risk compared to those calving between 24 and 28 mo, attributing late calvings to issues in herd management, fertility, health problems, and increased rearing costs (Sewalem et al., 2005). Understanding these relationships could improve breeding and management strategies aimed to increase herd longevity.

In dairy cattle, authors have observed positive genetic correlations among body traits and longevity and a strong relationship with functional herd life (Hu et al., 2021). In beef cattle, Forabosco et al. (2004) discovered that animals with higher muscle development were more likely to remain in the herd, indicating a phenotypic relationship between conformation and longevity. Body traits are routinely measured in Italian Charolais and Limousine herds and are part of the national breeding selection goals. However, to date, no studies have investigated the genetic association of conformation, fertility, and STAY traits in these breeds through a multiple-trait analysis.

Finally, despite numerous studies estimating heritability and genetic components in beef and dairy cattle, there is still a dearth of research on utilizing single-step genomic best linear unbiased prediction (**ssGBLUP**) approaches for consecutive calvings in beef cattle. Therefore, the objectives of this study were to 1) estimate the genetic parameters of STAY at different calving using a ssGBLUP approach, both Gaussian-linear and threshold, on Italian Charolais and Limousine beef cattle; 2) to compare Gaussian-linear and threshold models for STAY at different calvings of Limousine and Charolais, determining which approach provides a more accurate estimation of the genetic parameters of STAY; and 3) to estimate and to examine the genetic relationship between STAY and other traits, to identify potential indicators of longevity or to assess the impact of selection for STAY on other economically important traits and improve fertility on beef cattle.

## Materials and Methods

Phenotypic records, pedigree, and genomic information were provided by The National Italian Association of Limousine and Charolais Breeders database; therefore, Animal Care and Use Committee approval was unnecessary.

In Italy, the National Herd Book currently lists 11,579 registered Charolais and 50,511 Limousine females, highlighting their significant role in the Italian beef industry. Originating from France, the Limousine and Charolais breeds are extensively exported to numerous countries worldwide, either in purebred form or as part of crossbreeding systems (Bouquet et al., 2011).

The initial dataset for Limousine and Charolais breeds in this study comprises 587,773 calving records involving 141,923 cows born between 1977 and 2023. These records originate from 3,387 registered herds located across the entire national territory.

### Stayability traits

The trait STAY was chosen as a metric for assessing the longevity of animals, evaluated from the second to the eighth calving. By utilizing information on calving dates and order, seven cumulative STAY traits were assigned to each cow. These traits were defined as binary indicators of survival up to a specific calving order (e.g., survival up to the second calving, survival up to the third calving, and so on). Thus, each cow could have up to seven STAY records corresponding to survival up to the second through eighth calving (e.g., STAY2, STAY3, …, STAY8). Consequently, each female could have up to seven different observations if she reached STAY8. For each parity, STAY was treated as a binary trait, where a value of “1” indicated cows with a recorded calving event and “0” indicated those without a calving record. Consequently, STAY (1 = success, 0 = failure) was assigned based on the presence of a calving date for each parity up to parity eight. The study also considered animals that remained in the herd from one calving to the next “STAY to STAY traits” (traits STAY23, STAY34, …, STAY78), focusing solely on cows that survived from, for example, second to third calving. For instance, STAY23 measures survival from the second calving to the third, whereas STAY34 measures survival from the third calving to the fourth. Only cows that have reached the earlier calving event (e.g., second calving for STAY23) are included in these analyses. The inclusion of these traits in the model (e.g., STAY2 with STAY23, or STAY3 with STAY34), allowing for a more complete dataset by including cows that may not have reached the next calving.

Initial data editing involved keeping only females with age at first calving (**AFC**) between 700 and 1,400 d of age. Cows with recorded calving events and with an appropriate interval before the following calving, allowing for the occurrence or absence of the subsequent calving event, were included in the analysis. Specifically, animals with any calving intervals shorter than 290 d or longer than 550 d were excluded from the dataset. Twins’ parities were considered as a single calving event. If the time elapsed between the last recorded calving of a cow and the date when the calving data was extracted was less than 550 d, any calving records that occurred after this period were classified as censored data. This means that these records were not fully available for analysis because the full calving history was incomplete by the time of data extraction. Censored records were treated as missing data in the model (Smith 1990; Stephen et al., 2023). Additionally, cows with missing sire and dam were removed. Supplementary Table S1 reported the percentage of censored records according to the total number of cows for all the STAY for the two breeds.

The final dataset included 47,362 cows, of which 38,188 were daughters of 6,194 sires belonging to 2,471 herds for Limousine, and 9,174 were daughters of 1,774 sires belonging to 712 herds for Charolaise, respectively. Table 1 shows the number of records for all the STAY and the relative incidence of survival for the two breeds.

**Table 1.** Number of the cows and incidence to survival according to the number of records per each of the stayability (STAY)^1^ for Limousine and Charolaise

Trait	Definition	Limousine			Charolais		
		*N* ^2^	*N* of cow survived	Incidence (survival %)	*N* ^2^	*N* of cow survived	Incidence (survival %)
STAY1	Stayability as a first parity = 1; failed = 0	38,188	38,188	100.00	9,174	9,174	100.00
STAY2	Stayability as a second parity = 1; failed = 0	33,209	25,526	66.84	8,071	5,557	60.57
STAY3	Stayability as a third parity = 1; failed = 0	30,105	18,764	49.14	7,519	3,913	42.65
STAY4	Stayability as a fourth parity = 1; failed = 0	27,978	14,381	37.66	7,078	2,820	30.74
STAY5	Stayability as a fifth parity = 1; failed = 0	26,546	11,257	29.48	6,774	2,041	22.25
STAY6	Stayability as a sixth parity = 1; failed = 0	25,372	8,838	23.14	6,550	1,467	16.00
STAY7	Stayability as a seventh parity = 1; failed = 0	24,429	6,890	18.04	6,381	1,042	11.36
STAY8	Stayability as a eight parity = 1; failed = 0	23,657	5,314	13.91	6,245	717	7.82

^1^Continuity in herd until the subsequent parity (Success = 1; Failure = 0).

^2^Total number of cows, including both those that survived and were culled.

### Fertility and conformation traits

Data on fertility traits were extracted from records of cows from the STAY dataset. Fertility traits considered were first calving interval (**FCI**, days) and AFC (days), and were treated as continuous responses. Values of FCI averaged 405.7 d (± 60.87 SD) and 401.50 (±60.05 SD) for Limousine and Charolais, respectively. Values of AFC averaged 1,026 d with an SD of 143.69 and 1,026 d with an SD of 157.62 for Limousine and Charolais, respectively.

Conformation-type traits in the two breeds are routinely measured once in the lifetime, typically around weaning and yearling (between 6 and 15 mo of age). Conformation traits were evaluated for each animal by trained classifiers between 1990 and 2023 and were obtained by visual scores. Muscularity refers to the muscle development of the animal across several reference sites, including the shoulder, loin, rump, and hindquarter (Boligon et al., 2011). In this study, we focused on eight traits related to conformation and muscularity traits. These traits assess muscular development, specifically the musculature of the thigh and back, as well as skeletal development, which measures proportionality. Additionally, traits like rump length and overall size capture general body development. Traits were assessed using scores ranging from 1 to 10, with 10 representing the highest expression of the trait and 1 indicating the lowest expression. Conformation traits considered were wither width, convexity and length of rump, length of dorsolumbar line, rear and back width, pelvic length, and development. The final dataset consisted of 32,119 and 7,537 measured cows for Limousine and Charolais, respectively. The number of records, the mean, and the definition per each conformation trait are reported in Supplementary Table S2.

### Pedigree and genotypes

The raw pedigree files included 147,801 and 526,887 animals. For subsequent analyses, animals were traced back eight generations for Charolais and six generations for Limousine, resulting in 13,222 and 35,418 animals, respectively.

In total, 276 and 71 animals were genotyped with a panel of 119,854 single nucleotide polymorphism (**SNP**) (GeneSeek GGP Bovine 150K; Illumina Inc., San Diego, CA) for Limousine and Charolais, respectively. 3,745, and 1,047 individuals were genotyped with a panel of 28,299 SNPs (GeneSeek GGP Bovine LD v3; Illumina Inc.) for Limousine and Charolais, respectively. All SNPs were mapped according to ARS-UCD1.2 genome assembly (Rosen et al., 2020). After the mapping, 118,135 markers for GeneSeek GGP Bovine 150K and 27,654 markers for GeneSeek GGP Bovine LD v3 were considered. The two panels shared 13,984 SNPs. Animals genotyped with GeneSeek GGP Bovine LD v3 were imputed to GeneSeek GGP Bovine 150K, leveraging the common set of 13,984 SNPs shared between the two panels as a basis for alignment and enhancement. Subsequently, the 13,670 nonoverlapping SNPs were imputed, and the resulting genotypes were integrated with the previously imputed set of 118,135 SNPs. This process led to a total of 131,805 imputed SNPs by the conclusion of the last step. FImpute v.3 (Sargolzaei et al., 2014) was utilized with default parameters for genotype imputation. The genotype imputation was performed separately for each breed, with genotypes for the Charolais and Limousine breeds being imputed independently.

Quality control (**QC**) and genotype data filtering were performed using PLINK v1.9 (Chang et al., 2015). Autosomal SNPs and individuals with more than 10% missing values, minor allele frequencies lower than 1%, and a call rate lower than 90% were excluded during QC. After QC, 116,221 SNPs and 3,960 animals remained for Limousine, while 116,704 SNPs and 990 animals remained for Charolais. Table 2 summarizes the number of genotyped animals per category, including females with phenotype, sires, and dams.

**Table 2.** Number of genotyped animals for Charolaise and Limousine considering animals with phenotype, sires, and dams.

Breed	GeneSeek GGP Bovine LD v3			GeneSeek GGP Bovine 150K			Total
	Female with phenotype^1^	Sires^2^	Dams^3^	Female with phenotype^1^	Sires^2^	Dams^3^	
Charolais	504	274	182	10	52	6	1,028
Limousine	2,233	906	651	256	106	56	4,054

^1^Female with phenotype are those individuals showing a phenotypic record in the dataset. They may appear as dams or other individuals.

^2^Male as sires without phenotype are those individuals not showing a phenotypic record in the dataset, but they appear as sires of individuals with phenotypes.

^3^Female as dame without phenotype are those individuals not showing a phenotypic record in the dataset, but they appear as dams of individuals with phenotypes.

### Model computation and analysis

#### Univariate models

In our analysis, an underlying continuous variable termed liability was derived from the data based on the following assumption:


$$\begin{array}{l} y=0,\;if\;\lambda \leq \tau \;and\;y=1,\;if\;\lambda >\tau \end{array}$$

where *y* represents the linear observation (0/1), λ denotes the liability, and τ stands for the specified threshold. Each observation of *y* assumes a value of 1 (indicating success in herd until the subsequent parity) when the liability surpasses *τ*, and 0 (reflecting failure in the herd on the subsequent parity) otherwise. The liability is assumed to follow a normal distribution with mean *µ* and covariance matrix $R=I\Sigma_{\lambda }^{2}$, where $\Sigma_{\lambda }^{2}$ denotes the variance of the scale. The liability is assumed to follow a normal distribution with mean µ and variance $\;\Sigma_{\lambda }^{2}$. Given that the mean and variance of the liability are nonidentifiable, we set this parameter to $\Sigma_{e}^{2}=1$ and τ=0. Consequently, there is no necessity for sampling the threshold value.

STAY were analyzed fitting a univariate threshold and Gaussian-linear animal model. Genetic parameters were estimated for STAY2 to STAY8 using the following models:


$$\begin{array}{l} MODEL\;\;1:\;y\;=\;X_{\beta }\;+\;Z_{p}p\;+\;Z_{a}a+e \end{array}$$

$$\begin{array}{l} MODEL\;\;2:\;\lambda \;=\;X_{\beta }\;+\;Z_{p}p\;+\;Z_{a}a\;+\;e \end{array}$$

where *y* is the linear vector of STAY traits (0/1) and λ the unobserved liability for threshold models; **β** is the vector associated to fixed effects of the year of calving (34 and 48 levels for Charolais and Limousine, respectively); *p* is the vector associated to the random effect of herd; *a* is the vector for the animal additive genetic effect; *e* is the vector associated to the random residual error; and **X**, **Z**_**p**_**, Z**_**a**_ are the incidence matrices related to fixed, random, and additive genetic effect of the animal, respectively. The solutions for additive genetic effects were assumed as $a∼N\left(0,H\Sigma_{a}^{2}\right)$, where **H** is the relationship matrix and $\Sigma_{a}^{2}$ the additive genetic variance and the residual error values are assumed as $e∼\;N\left(0,I\Sigma_{e}^{2}\right)$, where ***I*** is the identity matrix and $\Sigma_{e}^{2}$ is their variance.

The SNPs informations among animals was included in the form of a genomic relationship matrix. Therefore, the **H** matrix was constructed by combining the pedigree relationship matrix (**A**) and an SNP-derived genomic relationship matrix (**G**) (Legarra et al., 2014) and was estimated through ssGBLUP as described by Aguilar et al. (2010). The **G** matrix was built using the second method proposed by VanRaden (2008).

#### Multivariate models

Multivariate models enable the simultaneous analysis of several STAY traits as correlated traits and can be utilized for STAY to specific endpoints or within time intervals. Genetic correlations between the traits were estimated by using bivariate threshold models as follows:


$$\begin{array}{l} MODEL\;\;3:\;\left[\begin{array}{l} \lambda_{1}\;\lambda_{2}\; \end{array}\right]\;=\;\left[\begin{array}{l} X_{1}\;0\; \end{array}\begin{array}{l} \;0\;X_{2}\; \end{array}\right]\left[\begin{array}{l} \beta_{1}\;\beta_{2}\; \end{array}\right]+\;\left[\begin{array}{l} Z_{p1}\;0\; \end{array}\begin{array}{l} \;0\;Z_{p2}\; \end{array}\right]\left[\begin{array}{l} p_{1}\;p_{2}\; \end{array}\right]\;+\;\left[\begin{array}{l} Z_{a1}\;0\; \end{array}\begin{array}{l} \;0\;Z_{a2}\; \end{array}\right]\left[\begin{array}{l} a_{1}\;a_{2}\; \end{array}\right]\;+\;\left[\begin{array}{l} e_{1}\;e_{2}\; \end{array}\right]\; \end{array}$$

where $\lambda_{1}$ is the unobserved liability for STAY observations (STAY2, …, STAY8), and $\lambda_{2}$ is the unobserved liability for STAY23 until STAY78; fixed and random effects were the same as in the univariate analyses (models 1 and 2). Additionally, a bivariate model was applied also for fertility traits, and FCI and AFC were treated as continuous variables using the same fixed and random effect. Censored records were treated as missing and not included in the analyses. In particular, the calving season for STAY23 refers to the period when a cow gives birth, marking the beginning of its second calving. This is the time frame during which the cow gives birth to its second calf and transitions into its third parity. As reported by Hardie et al. (2021), bivariate models were used to address possible selection bias on STAY23, as only cows that survived to their second parity had data for the STAY2 trait. Including STAY2 along with STAY23 in the analysis, it was possible to consider traits from all cows in the herd that fit the criteria reducing any bias that might occur using a nonrandom selection of cows that reached their third parity.

Genetic correlations among conformation and STAY traits were estimated with the following bivariate threshold model:


$$\begin{array}{l} MODEL\;4:\;\left[\begin{array}{l} \lambda \;y\; \end{array}\right]\;=\;\left[\begin{array}{l} X_{1}\;0\; \end{array}\begin{array}{l} \;0\;X_{2}\; \end{array}\right]\left[\begin{array}{l} \beta_{1}\;\beta_{2}\; \end{array}\right]\;+\;\left[\begin{array}{l} Z_{p1}\;0\; \end{array}\begin{array}{l} \;0\;Z_{p2}\; \end{array}\right]\left[\begin{array}{l} p_{1}\;p_{2}\; \end{array}\right]\;+\;\left[\begin{array}{l} Z_{w1}\;0\; \end{array}\begin{array}{l} \;0\;Z_{w2}\; \end{array}\right]\left[\begin{array}{l} 0\;w_{2}\; \end{array}\right]\;+\;\left[\begin{array}{l} Z_{a1}\;0\; \end{array}\begin{array}{l} \;0\;Z_{a2}\; \end{array}\right]\left[\begin{array}{l} a_{1}\;a_{2}\; \end{array}\right]\;+\;\left[\begin{array}{l} e_{1}\;e_{2}\; \end{array}\right] \end{array}$$

where λ the unobserved liability for STAY observations (STAY2, …, STAY8), and *y* is the linear vector of the conformation trait; β_1_ and β_2_ are the vectors associated to fixed effects of the year of calving and age of the cow at the evaluation; *p*_1_ and *p*_2_ are the vectors associated to the random effect of herd; *w*_2_ is the vector associated to the random effect of the code of the trained classifiers; *a*_1_ and *a*_2_ are the vectors for the animal additive genetic effect; *e* is the vector associated to the random residual error; and **X**, **Z**_**w**_, **Z**_**a**_ are the incidence matrix related to fixed, random, and additive genetic effect of the animal, respectively. The random effect of the trained classifiers correlates and covaries only with the conformation traits, as only these traits are assessed by the classifiers.

In particular, the assumptions regarding the (co)variance structure in the bivariate models of additive genetics, the random effect of herd and trained classifiers, and residual effects were estimated using the following matrix notations:


$$\begin{array}{l} G=\left|\begin{array}{c} \;\Sigma_{a1}^{2}\;\Sigma_{a2a1}\; \end{array}\begin{array}{c} \;\Sigma_{a1a2}\;\Sigma_{a2}^{2}\; \end{array}\right|;P=\left|\begin{array}{c} \;\Sigma_{p1}^{2}\;\Sigma_{p2p1}\; \end{array}\begin{array}{c} \;\Sigma_{p1p2}\;\Sigma_{p2}^{2}\; \end{array}\right|;W=\left|\begin{array}{c} 0\;0\; \end{array}\begin{array}{c} \;\;\;0\;\Sigma_{w2}^{2}\; \end{array}\right|;R=\left|\begin{array}{c} \Sigma_{e1}^{2}\;0\; \end{array}\begin{array}{c} \;0\;\Sigma_{e2}^{2}\; \end{array}\right| \end{array}$$

where **G** is the matrix of additive genetic (co)variances $\Sigma_{a1}^{2}$, $\Sigma_{a1a2}$, and $\Sigma_{a2}^{2}$ of traits one and two, **P** and **W** are matrices of (co)variances of random effects $\Sigma_{p1}^{2}$, $\Sigma_{p1p2}$, $\Sigma_{p2}^{2}$, and $\Sigma_{w2}^{2}$, respectively, of traits one and two, and **R** is the matrix of residual (co)variances $\Sigma_{e1}^{2}$ and $\Sigma_{e2}^{2}$ of traits one and two.

### Genetic parameters

The (co)variance components were estimated using the Gibbs sampling algorithm implemented in the BLUPF90 family software (Aguilar et al., 2018). Threshold models were run using THRGIBBS1F90, and Gaussian-linear models were run using GIBBS3F90 programs. All analyses were run for 100,000 cycles with a burn-in of 50,000 samples and every 10th sample being stored, for a total of 5,000 samples used for subsequent inference. Convergence was assessed by visual inspection of trace plots and throughout Geweke’s test using the package “coda” (Plummer et al., 2006).

Heritability (*h*^2^), herd effect (*h*_*i*_), and intra-herd heritability ($h_{IH}^{2}$) are expressed as follows:


$$\begin{array}{l} h^{2}\;=\;\frac{\Sigma_{a}^{2}}{\Sigma_{a}^{2}\;+\;\Sigma_{p}^{2}\;+\;\Sigma_{e}^{2}} \end{array}$$

$$\begin{array}{l} h_{i}\;=\;\frac{\Sigma_{p}^{2}}{\Sigma_{a}^{2}\;+\;\Sigma_{p}^{2}\;+\;\Sigma_{e}^{2}} \end{array}$$

$$\begin{array}{l} h_{IH}^{2}\;=\;\frac{\Sigma_{a}^{2}}{\Sigma_{a}^{2}\;+\;\Sigma_{e}^{2}} \end{array}$$

where $\Sigma_{a}^{2}$, $\Sigma_{p}^{2}$, and $\Sigma_{e}^{2}$ are the variance components related to additive genetic effect, random effect of the herd, and random residual error. Lower and upper limits of the 95% highest posterior probability distributions and the means for heritabilities were estimated from the Gibbs samples. Confidence intervals and posterior means were used as estimates and its attached standard error.

The study compared univariate analyses, incorporating both threshold and Gaussian-linear models, to examine the genetic relationship among STAY traits. To assess the possibility of re-ranking among genotyped sires, Spearman’s rank correlations were estimated. Specifically, ssGEBV from univariate models for STAY2 through STAY8 were compared for both Charolais and Limousine breeds. Additionally, the study evaluated ssGEBVs for genotyped sires to verify their average genetic superiority and to determine whether any loss in expected progeny differences would occur if selection were based on STAY traits. For example, a reduction in the average genetic superiorities in STAY2 would be expected if the selection was based on STAY8.

The genetic correlation among traits in bivariate models was obtained as follows:


$$\begin{array}{l} r_{gen}\;=\;\frac{\Sigma_{a,xz}}{\sqrt{\Sigma_{a,x}^{2}\;\times \;\Sigma_{a,z}^{2}}} \end{array}$$

where $\Sigma_{a,xz}$ is the genetic covariance between the two considered traits in the bivariate analysis, and $\Sigma_{a,x}^{2}$ and $\Sigma_{a,z}^{2}$ are the additive genetic variances, respectively.

## Results and Discussion

Linear mixed models provide enhanced flexibility for modeling binary traits, even when the assumptions are violated. This preference is attributed to several factors, including the flexibility of linear mixed models in handling various types of data. They can handle both continuous and categorical predictors, making them suitable for a wide range of analyses. Furthermore, their ease of interpretation through understandable coefficients for fixed effects and their efficiency in providing unbiased estimates of parameters even when data violate assumptions (Breslow and Clayton, 1993). Despite this, both threshold and linear mixed models’ approaches were assessed in this study to gain comprehensive insights into STAY traits in two different beef cattle breeds.

### Incidence of survival

Table 1 shows the incidence of survival of STAY2 until STAY8 in the Limousine and Charolais. The cull rate of primiparous cows between the first and second STAY was 33% and 40% for Limousine and Charolais, respectively. The cull rate until STAY8 was 86% and 92% for Limousine and Charolaise, respectively. Culled animals were higher in early STAY than those between later STAY and relatively similar among breeds. On average, survival between parities was 6% to 7% lower in Charolais compared to Limousine.

The elevated culling rate observed in early STAY can be attributed to several factors. First, primiparous cows often encounter reproductive challenges, such as calving difficulties or postpartum complications, which can lead to their removal from the herd. Additionally, some cows may exhibit inadequate maternal instincts or abilities, resulting in lower calf survival rates and subsequent culling. Furthermore, heifers that fail to initiate a first parity may be sold for meat purposes. Genetic selection also plays a role, as genetically superior females are retained in the herd, leading to the culling of less desirable individuals. These reproductive challenges, maternal abilities, and genetic selection pressures collectively contribute to the higher culling rate observed during early calving.

Martinez et al. (2005) investigated STAY traits from calving 2 to 6 in Hereford cows, while Silva et al. (2018) compared the same traits in Guzerá, Nelore, and Tabapuã cattle. Both studies found similar culling rates for early calving, consistent with the present study’s findings.

### Heritability

**Figure 1.**
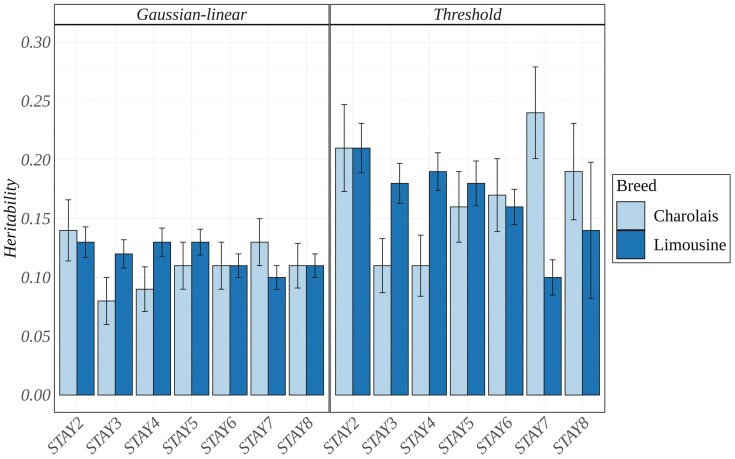
Heritability estimates with the relative SE across stayability (STAY) traits for Limousine and Charolais using a single-step GBLUP approach considering STAY (Gaussian-linear model) and liability of STAY (threshold model), respectively.

Estimates of heritability and their relative SE for STAY using threshold and Gaussian-linear ssGBLUP models are summarized in Figure 1. Generally, the heritability estimates using the Gaussian-linear model were low, ranging from 0.13 ± 0.01 for STAY2 to 0.11 ± 0.01 for STAY8 in Limousine cattle and from 0.14 ± 0.03 for STAY2 to 0.11 ± 0.02 for STAY8 in Charolais cattle. In contrast, heritability values were moderate when using the threshold model, ranging from 0.21 ± 0.02 for STAY2 to 0.14 ± 0.06 for STAY8 in Limousine and from 0.24 ± 0.04 for STAY2 to 0.19 ± 0.04 for STAY8 in Charolais. When considering the Gaussian-linear model, the trends for heritability were similar between the two breeds. However, the threshold model showed a slightly different trend in Limousine cattle, decreasing heritability with increasing parities.

Estimates of heritability using the Gaussian-linear model in our study are consistent with those obtained using linear random regression models for STAY in late calvings (0.12) (Jamrozik et al., 2013). Still, they are lower than the estimates for STAY in early calvings (0.38) reported for Canadian Simmental cattle (Jamrozik et al., 2013). For Guzerá, Nelore, and Tabapuã cattle, Silva et al. (2018) used a linear random regression model and, when transforming the heritabilities to the underlying normal scale, found estimates for the first calving number similar to our results but lower estimates for later calving numbers. In Hereford cattle, Martinez et al. (2005) used linear binomial models and found heritability estimates ranging from 0.18 ± 0.09 to 0.25 ± 0.07 for STAY to consecutive calving. These results were generally higher compared to our study.

Estimates of heritability using the threshold model in our study were, on average, slightly lower than the heritability estimate for STAY to consecutive calvings (0.24) on the underlying liability scale in Simmental cattle (Jamrozik et al., 2013). Santana et al. (2012) reported a heritability of 0.11 using a threshold model in Nelore cattle, which is similar to our estimates for STAY2 and STAY3 in Charolais and STAY7 in Limousine cattle. Our estimates were generally lower than the heritability values (0.25, 0.22, and 0.28) for STAY at specific ages in Nelore cattle provided by Van Melis et al. (2007). Martinez et al. (2005) used a threshold model in Hereford cattle and found heritability estimates from 0.29 ± 0.10 to 0.39 ± 0.11 for STAY to consecutive calving, the results from our study were generally lower in comparison.

In our analysis, as shown in Figure 1, we found that the heritability estimates obtained through a Gaussian-linear model were lower than those obtained through the threshold model, as also reported by Martinez et al. (2005). These moderate estimates of heritability for STAY imply the potential for a response to selection and genetic improvement through genetic selection. These findings underscore the utility of utilizing STAY as a selection criterion to enhance dam fertility. Supplementary Table S3 reported the numerical values of heritabilities with their respective HPDI. In addition, variance components and the corresponding HPDI considering threshold and Gaussian-linear model were reported in Supplementary Table S4.

### Intra-herd heritability and herd effect

In genetic analyses, estimation of herd effect and intra-herd heritability help to understand the balance between genetic and environmental influences within herds. Similar values between intra-herd heritability and overall heritability suggest that the herd genetic variability and environmental conditions are representative of the studied population. Conversely, large differences indicate significant environmental impacts, which could mask genetic variation. Higher herd effect estimates imply substantial environmental influence, with high variability in management practices, nutrition, and health care. This variability can slow genetic progress for the analyzed trait.

Estimates of the herd effect and relative SE are reported in Supplementary Figure S1. Estimates were moderate for both Charolais and Limousine breeds. Using the Gaussian-linear model, herd effect values ranged from 0.25 ± 0.01 for STAY2 to 0.15 ± 0.01 for STAY8 in Limousine and from 0.20 ± 0.02 for STAY2 to 0.12 ± 0.01 for STAY8 in Charolais. Using the threshold model, herd effect values ranged from 0.34 ± 0.01 for STAY2 to 0.56 ± 0.09 for STAY8 in Limousine and from 0.30 ± 0.03 for STAY2 to 0.45 ± 0.04 for STAY8 in Charolais.

While intra-herd heritability focuses on genetic variation within a herd, the herd effect considers the impact of environmental factors common to all animals within the same group. These environmental factors include management practices, feeding regimes, housing conditions, disease exposure, and social interactions. The herd effect can significantly influence the observed phenotypic variation of traits within a herd, independent of genetic differences among individuals.

As reported Supplementary Figure S1, both models for both breeds exhibited an opposite trend: increasing for the Gaussian-linear model and decreasing for the threshold model. For both analyses, Limousine showed, on average, slightly higher values than Charolais of herd effect with significative differences in STAY2 using the Gaussian-linear model and STAY7 using the threshold model. The observed differences in herd effect estimates between the Charolais and Limousine breeds for STAY may be attributed to several factors. Genetic differences between the breeds could influence their respective responses to herd environments, with varying levels of resilience or susceptibility to environmental factors affecting longevity. Additionally, distinct management practices specific to herds, like feeding, housing, and herd management, could impact the two breeds differently. Environmental adaptability may also play a role, as one breed might be better suited to certain conditions or practices, thereby explaining the higher herd effect values observed in Limousine.

Estimates of intra-herd heritability were summarized in Supplementary Figure S2, showing lower to moderate estimations for Charolais and Limousine. Using the Gaussian-linear model, values ranged from 0.18 ± 0.02 for STAY2 to 0.13 ± 0.01 for STAY8 considering Limousine and 0.17 ± 0.03 for STAY2 to 0.12 ± 0.02 for STAY8 considering Charolais. Using the threshold model, intra-herd heritability showed values ranged from 0.32 ± 0.03 for STAY2 to 0.33 ± 0.04 for STAY8 and ranged from 0.30 ± 0.05 for STAY2 and 0.34 ± 0.06 for STAY8 for Limousine and Charolais, respectively. Intra-herd heritability refers to the proportion of the phenotypic variance of a trait attributable to genetic differences among individuals within a specific group or herd. The fraction of observed trait variability within a given population could be attributed to genetic variation among individuals within that population, excluding the effect of common environments shared by animals within the same group. Thus, intra-herd heritability provides an estimate of the heritability of a trait within a specific selection unit, such as a herd. In this study, the intra-herd heritability values were, on average, similar to the heritability, suggesting that the genetic potential for the trait within the herd is consistent with the population.

As reported in Supplementary Figure S2, both models for both breeds exhibited a trend comparable to that of heritabilities described in the previous section, with higher values detected using the threshold model. For both analyses, Limousine showed slightly higher average values compared to Charolais, with significant differences in STAY3, STAY4, and STAY5 using both Gaussian-linear and threshold models. Only for STAY7 did Charolais show significantly higher values compared to Limousine.

These differences between the two breeds may arise due to various factors. If one breed exhibits greater genetic variability for functional longevity compared to the other, a higher degree of intra-herd heritability may be observed in that breed. Disparities in rearing environments, management practices, and selection procedures between the two breeds might impact STAY and the estimation of intra-herd heritability. Differences in climate and nutrition could influence animal survival and fertility, thus affecting the estimation of trait intra-herd heritability. Additionally, if one breed is more susceptible to genetic-environmental interactions affecting functional longevity, a reduction in intra-herd heritability may be observed in that breed compared to the other. Numerical values for intra-herd heritabilities and herd effect with their respective HPDI are reported in Supplementary Table S3.

### Re-ranking among genotyped sires

We investigated whether different models (threshold vs. Gaussian-linear) would significantly re-rank genotyped sires, potentially impacting selection decisions based on ssGEBV for functional longevity. Only genotyped sires were considered for both Charolais and Limousine cattle.

Analyzing regressions coefficients and Spearman correlations among the genotyped sire for STAY2 on the sires ssGEBV for STAY3 and STAY8 could allow us to verify whether there would be loss in terms of expected progeny difference and re-ranking of sires across different calvings if sire selection were based on early or late longevity. A reduction in the average genetic superiorities for sires in STAY2 would be expected if the selection were based on the more distant calving event, for example, STAY8. For the Limousine breed, the regression coefficients between STAY2 and STAY3 were 0.80 for the threshold model and 0.74 for the Gaussian-linear model, while between STAY2 and STAY8, the coefficients were 0.34 and 0.33, respectively. Similarly, for the Charolais breed, the regression coefficients between STAY2 and STAY3 were 0.86 for the threshold model and 0.83 for the Gaussian-linear model, and between STAY2 and STAY8, the coefficients were 0.21 and 0.32, respectively. In addition, to understand the extent of re-ranking between STAY2 with early (STAY3) and late (STAY8) calvings were analyzed the top 100 sires based on their genomic values. By tracking their positions for STAY3 and STAY8, the changes in rankings were calculated, and the average re-ranking was determined. The average accuracy for sires in the Charolais breed was 0.24, 0.16, and 0.15 for STAY2, STAY3, and STAY8, respectively, using a Gaussian-linear model, and 0.45, 0.36, and 0.27 for STAY2, STAY3, and STAY8, respectively, using a threshold model. For the Limousine breed, the average accuracy for sires was 0.33, 0.29, and 0.22 for STAY2, STAY3, and STAY8, respectively, with a Gaussian-linear model, and 0.53, 0.51, and 0.39 for STAY2, STAY3, and STAY8, respectively, with a threshold model.

**Figure 2.**
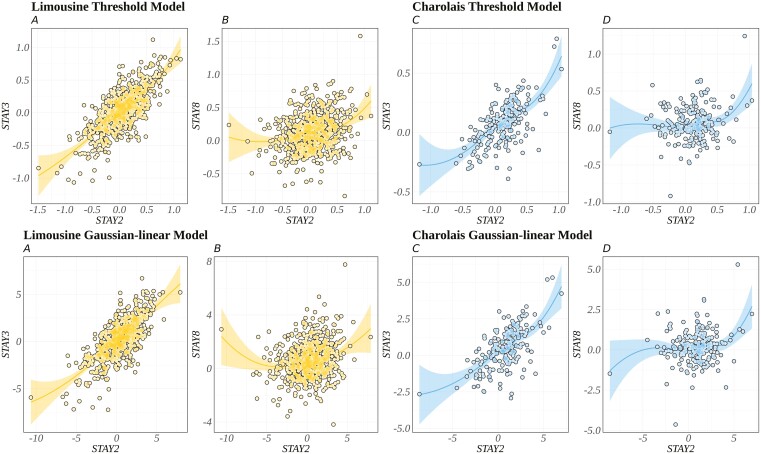
Regression coefficient for stayability (STAY) traits, more in specific between STAY2 with STAY3 and STAY8 among solutions for genotyped sires considering threshold and Gaussian-linear models for both breeds. Threshold and Gaussian-linear model for Limousine, respectively (A, B). Threshold and Gaussian-linear model for Charolais, respectively (C, D).

In both the Limousine and Charolais breeds, analyses using both Gaussian-linear and threshold models exhibited similar patterns (Figure 2), with a reduction in the regression coefficient between STAY2 and STAY8. Additionally, Spearman correlations among genotyped sires were assessed. For the Limousine breed, strong correlations between STAY2 and STAY3 were observed, with values of 0.70 for both the threshold and Gaussian-linear models. In contrast, the Charolais breed displayed moderate correlations, with values of 0.57 and 0.58 for the threshold and Gaussian-linear models, respectively. Lower correlations were found for both breeds when comparing STAY2 with STAY8. Specifically, Spearman’s rank correlations were 0.24 and 0.20 for the Limousine breed using the threshold and Gaussian-linear models, respectively. For the Charolais breed, these values were 0.12 and 0.10 using the threshold and Gaussian-linear models, respectively.

The average re-ranking of sires between STAY2 and subsequent calvings revealed similar trends. In the Charolais breed, the average re-ranking was 26 positions for early (STAY3) and 32 for late (STAY8) calvings. For the Limousine breed, the average re-ranking was 21 positions for early (STAY3) and 31 for late (STAY8) calvings. These trends remained consistent across both models. Considering the regression coefficients from Figure 2 and the values from Spearman correlations among STAY traits, it appears that sires may be re-ranked across different parities, with more substantial re-ranking observed in more distant calvings. This potential re-ranking could lead to varying genetic gains. These findings suggest that STAY traits for different calvings represent distinct traits from a genomic perspective. Sires with high genomic values for STAY based on early calving may not necessarily maintain their performance in later calvings.

The reduction in the regression coefficient for STAY2 versus STAY8 implies that selecting sires based on STAY8 might result in smaller improvements in STAY2 compared to selecting STAY3. This indicates that selecting sires for longevity would primarily result in genetic gains for STAY up to the second calving. The observed average re-ranking for Charolais and Limousine beef cattle among the top 100 sires indicates variability in sire performance between early and late calvings. This variability has significant implications for breeding strategies. Breeders should consider the potential for re-ranking when selecting sires based on early calving performance to ensure sustained genetic improvement across multiple calvings.

While assessments of STAY2 may be more predictive of early calving (STAY3), the relationship between STAY2 and STAY8 is lower, suggesting a weaker relationship between early and late STAY (Figure 2). More precisely, selecting sires based on STAY8 may result in smaller improvements in STAY2 than selecting sires based on STAY3. In this context, re-ranking sires based on STAY8 may prioritize those with consistently high longevity over the long term. Identifying sires with consistently high genomic values for STAY across both early and late calving emphasizes the importance of selecting sires based on their capacity to maintain superior STAY performance over time. This could contribute to the continual enhancement of functional longevity traits within the herd.

Jamrozik et al. (2013) reported a decreased genetic and phenotypic correlation among different STAY to calving in Canadian Simmental cattle. Authors found positive values, and the magnitude of correlations decreased with the increasing distance between parities, confirming that more distance STAY was not the same traits. In Nelore cattle, some authors analyzed the regression coefficient of the sires EBV for STAY at 5 and 6 yr of age on the sires EBV for STAY at 7 yr of age. They verified a reduction in the average genetic superiorities and expected progeny difference in STAY at 7 yr of age if the selection was based on STAY at 5 and 6 yr of age (Van Melis et al., 2007).

One of the objectives of this study was to test the potential of Gaussian-linear models to be used in place of threshold-liability models, for the genetic evaluation of functional longevity. While a threshold model is theoretically the optimal choice for the analysis of these groups of traits, the Gaussian model is considered more straightforward because it requires less computational effort and is easier to interpret. The results indicate that when genetic parameter estimates and sire rankings are similar between the two approaches, the Gaussian model may be preferable due to its simplicity and fewer assumptions. Therefore, if both models yield comparable results, selecting the simpler model is often advantageous. However, the final choice depends on specific analytical requirements and the context of application, with the threshold model remaining a valid option when it better captures the discrete nature of the trait.

### Genetic correlation

**Figure 3.**
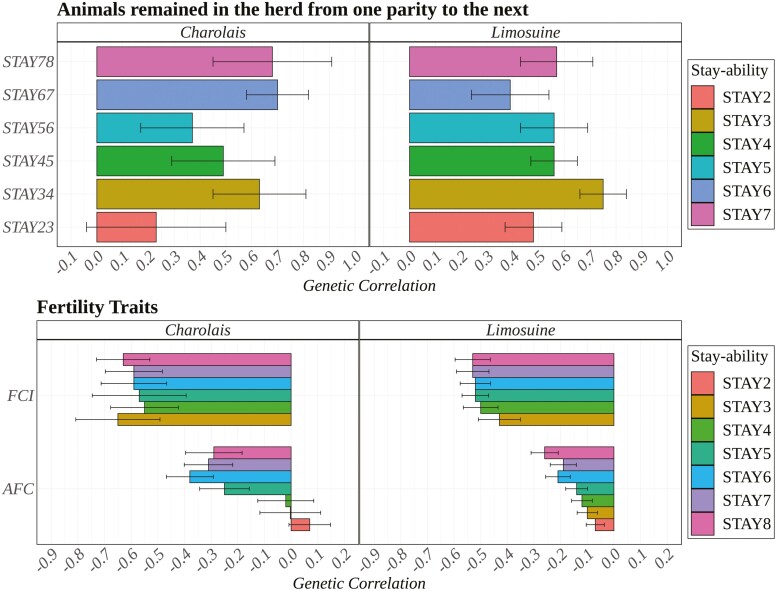
Genetic correlation between stayability (STAY) traits (STAY2 until STAY8) with fertility traits (FCI: first calving interval; AFC: age first calving) and animals remained in the herd from one parity to the next (STAY23 until STAY78), for Charolais and Limousine, respectively.

Genetic correlations were positive and strong for all STAY traits for Charolais and Limousine breeds (Figure 3). Specifically, the correlations between STAY2 and STAY3 were 0.48 for Limousine and 0.23 for Charolais. For STAY3 and STAY4, the correlations were 0.75 for Limousine and 0.63 for Charolais. Between STAY4 and STAY5, the correlations were 0.56 for Limousine and 0.49 for Charolais. For STAY5 and STAY6, the correlations were 0.56 for Limousine and 0.37 for Charolais. The correlations between STAY6 and STAY7 were 0.39 for Limousine and 0.70 for Charolais. Finally, the correlations between STAY7 and STAY8 were 0.57 for Limousine and 0.68 for Charolais.

These solid and positive genetic correlations indicate a consistent genetic basis for these traits across different parities. A study on Holstein cows in U.S. organic farms also reported strong and positive genetic correlations among STAY traits, yielding similar results to those in our study (Hardie et al., 2021).

Two fertility traits, AFC and FCI, were considered, and their genetic correlations with STAY, along with relative SE, are displayed in Figure 3. On average, these correlations were negative. For Limousine, AFC showed genetic correlations ranging from −0.07 for STAY2 to −0.26 for STAY8, with an increase in the negative correlation for later calvings. For FCI, moderate negative correlations were observed with STAY, with values ranging from −0.43 to −0.53 across different calvings. For Charolais, the genetic correlations for fertility traits were similar to those reported for Limousine. AFC exhibited a similar trend, with an increasing negative genetic correlation across parities, except for STAY2, STAY3, and STAY4, where the correlations were not significantly different from zero. FCI showed strong and stable negative genetic correlations with STAY, ranging from −0.65 for STAY2 to −0.63 for STAY8.

Given the definition of STAY, negative correlations between STAY and AFC and FCI were expected. Animals that begin breeding earlier and have shorter intervals between calvings are more likely to achieve longevity. Buzanskas et al. (2010) analyzed the genetic association between STAY and reproductive traits in Canchim beef cattle (Charolais × Zebu) and found a negative genetic correlation of −0.63 ± 0.20 with AFC. Similarly, a study on the genetic association of reproductive traits with longevity in Nellore cattle reported negative genetic correlations with STAY (Rizzo et al., 2015). Fertility traits such as AFC and FCI provide potential indirect indicators that could be used in early selection for longevity in cattle breeding programs.

**Figure 4.**
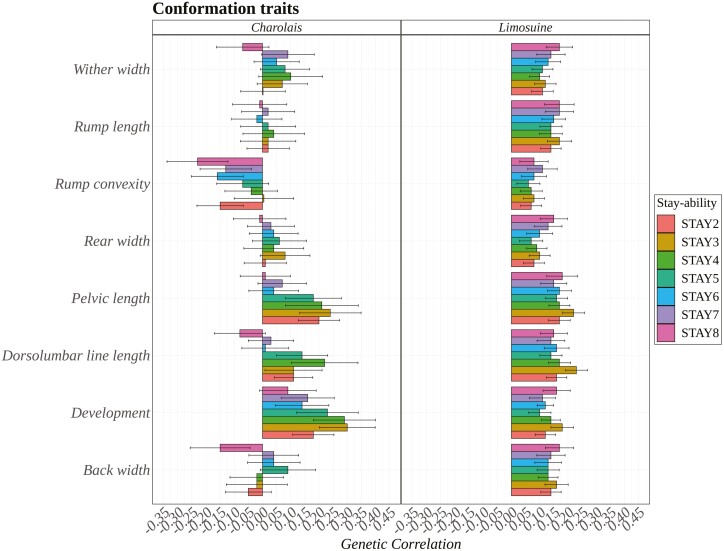
Genetic correlation between stayability (STAY) traits (STAY2 until STAY8) and eight conformation traits for Charolais and Limousine, respectively.

Figure 4 illustrates the genetic correlations between eight conformation traits and STAY traits for Limousine and Charolais. The analysis reveals notable differences between the two breeds in how these traits relate to cattle longevity from a genetic standpoint. For the Limousine breed, all correlations were positive and significantly different from zero. Mainly, traits such as pelvic length, dorsolumbar line length, development, and back width exhibited moderate genetic correlations with STAY traits, averaging above 0.20. This indicates that these specific conformation traits are likely to enhance the longevity of Limousine cattle due to their genetic influence.

In contrast, for the Charolais breed, many traits were essentially uncorrelated. This lack of significant correlation could be due to the fewer observations available for Charolais compared to Limousine cattle. Despite this, some conformation traits, such as pelvic length, dorsolumbar line length, and development, did show positive and significant correlations, mirroring the trends observed in Limousines. However, an exception was noted for rump convexity, which showed negative genetic correlations with STAY traits, suggesting that this particular trait may adversely affect the functional longevity of Charolais cattle. The observed differences in the relationship between longevity and conformation traits between Limousine and Charolais breeds could stem from various factors, including differences in herd sizes and genetic backgrounds. This variability highlights the importance of breed-specific strategies when selecting traits to improve cattle longevity.

Similarly to our results, many studies in the literature reported a positive genetic correlation between longevity and conformation linear type traits. Imbayarwo-Chikosi et al. (2018) and Sewalem et al. (2004) analyzed various traits, including body height, chest width, loin strength, rump angle, rump width, foot angle, bone quality, rear leg, and conformation, exhibited positive genetic correlations with longevity. In Chianina cattle, a study on phenotypic correlation revealed a consistent trend showing increased longevity in cows with greater muscle development across all muscularity traits. More specifically, Chianina cows with a long body and a deep and broad chest had a higher probability of survival; this suggests that cows exhibiting higher muscle development are more likely to remain in the herd (Forabosco et al., 2004). Similar to our findings for Charolais, which revealed negative genetic correlations with rump convexity, Buenger et al. (2001) observed a decrease in functional herd life for cows exhibiting highly ascending rumps. In contrast to our results, a study conducted in the Pirenaica beef breed emphasized a negative correlation between longevity and conformation, probably because individuals with higher muscularity could present calving difficulties (Varona et al., 2012).

## Conclusion

Longevity traits exhibited low to moderate heritability estimates using Gaussian-linear and threshold models. These heritability estimates for STAY suggest the potential for genetic improvement through selection. The moderate intra-herd heritability and herd effect estimation provide insights into the genetic and environmental factors influencing longevity within a herd. This knowledge enables breeders to enhance herd productivity and longevity through targeted breeding strategies. The regression coefficients, Spearman correlations, and average sire re-ranking observed in this study indicate significant re-ranking of sires between early and late calvings. Consequently, breeding strategies should account for these differences to ensure the selection of sires that maintain superior longevity traits across multiple calvings.

The strong positive genetic correlations for STAY across parities suggest that selecting longevity traits can be effective in improving average herd longevity. However, the negative associations between STAY and fertility traits, such as AFC and FCI, can provide as indirect indicators to select cows with greater potential for early longevity selection. Cows that begin breeding earlier and have shorter calving intervals have a higher probability of remaining in the herd. For Limousine cattle, the positive correlations between conformation traits and functional longevity suggest that animals with higher conformation scores may have greater longevity, which can enhance herd productivity.

Overall, these results suggest that making selection decisions based on animal conformation could increase cows’ robustness and resilience, thereby enhancing herd survival. Moreover, these findings emphasize the selection for longevity could optimize productivity and sustainability in beef cattle farming. Further studies and continued investigation into the genomic background of these traits could improve cattle longevity and identify potential genomic regions associated with genes affecting STAY in Limousine and Charolais breeds.

## Supplementary Data

Supplementary data are available at *Journal of Animal Science* online.

skae354_suppl_Supplementary_Tables_S1-S4_Figures_S1-S2

## Abbreviations:

AFC: age at first calving
ANACLI: The National Italian Association of Limousine and Charolais Breeders
FCI: first calving interval
ssGBLUP: single-step genomic best linear unbiased prediction
STAY: stayability

## Notes

This work was financially supported by Associazione Nazionale degli Allevatori delle razze bovine Charolaise e Limousine Italiane (Cup: J89H18000010005, https://www.anacli.it/i-beef-2). Grant was received by R.B. The funders had no role in study design and analysis, decision to publish, or manuscript preparation.

## Data and model availability statement

The datasets generated and/or analyzed during the current study are not publicly available due owned by a third party, ANACLI, Associazione Nazionale degli Allevatori delle razze bovine Charolaise e Limousine Italiane (http://www.anacli.it/) but are available from the corresponding author on reasonable request.

## References

[CIT0001] Aguilar, I., I. Misztal, D. L. Johnson, A. Legarra, S. Tsuruta, and T. J. Lawlor. 2010. Hot topic: a unified approach to utilize phenotypic, full pedigree, and genomic information for genetic evaluation of Holstein final score. J. Dairy Sci. 93:743–752. doi: https://doi.org/10.3168/jds.2009-2730 20105546

[CIT0002] Aguilar, I., S. Tsuruta, Y. Masuda, D. A. L. Lourenco, A. Legarra, and I. Misztal. 2018. BLUPF90 suite of programs for animal breeding with focus on genomics. In *Proceedings of the world congress on genetics applied to livestock production*, pp. 11–16.

[CIT0004] Boligon, A. A., M. E. Z. Mercadante, and L. G. Albuquerque. 2011. Genetic associations of conformation, finishing precocity and muscling visual scores with mature weight in Nelore cattle. Livest. Sci. 135:238–243. doi: https://doi.org/10.1016/j.livsci.2010.07.011

[CIT0005] Bouquet, A., E. Venot, D. Laloë, F. Forabosco, A. Fogh, T. Pabiou, K. Moore, J. Eriksson, G. Renand, and F. Phocas. 2011. Genetic structure of the European Charolais and Limousin cattle metapopulations using pedigree analyses. J. Anim. Sci. 89:1719–1730. doi: https://doi.org/10.2527/jas.2010-3469 21606443

[CIT0006] Breslow, N. E., and D. G. Clayton. 1993. Approximate inference in generalized linear mixed models. J. Stat. Data Sci. Educ. 88:9–25. doi:10.2307/2290687

[CIT0007] Buenger, A., V. Ducrocq, and H. H. Swalve. 2001. Analysis of survival in dairy cows with supplementary data on type scores and housing systems from a region of Northwest Germany. J. Dairy Sci. 84:1531–1541. doi: https://doi.org/10.3168/jds.S0022-0302(01)70187-7 11417714

[CIT0008] Buonaiuto, G., N. Lopez-Villalobos, A. Costa, G. Niero, L. Degano, L. M. E. Mammi, D. Cavallini, A. Palmonari, A. Formigoni, and G. Visentin. 2023. Stayability in Simmental cattle as affected by muscularity and body condition score between calvings. Front. Vet. Sci. 10:1141286. doi: https://doi.org/10.3389/fvets.2023.1141286 37065221 PMC10094164

[CIT0037] Buzanskas, M. E., D. A. Grossi, F. Baldi, D. Barrozo, D., Silva, L. O. C. Silva, R. A. A. T. Junior, D. P. Munari, and M. M. Alencar, 2010. Genetic associations between stayability and reproductive and growth traits in Canchim beef cattle. Livest Sci, 132:107-112. doi: https://doi.org/10.1016/j.livsci.2010.05.008.

[CIT0009] Chang, C. C., C. C. Chow, L. C. Tellier, S. Vattikuti, S. M. Purcell, and J. J. Lee. 2015. Second-generation PLINK: rising to the challenge of larger and richer datasets. GigaScience. 4:7. doi: https://doi.org/10.1186/s13742-015-0047-8 25722852 PMC4342193

[CIT0010] Forabosco, F., A. F. Groen, R. Bozzi, J. A. M. Van Arendonk, F. Filippini, P. Boettcher, and P. Bijma. 2004. Phenotypic relationships between longevity, type traits, and production in Chianina beef cattle1. J. Anim. Sci. 82:1572–1580. doi: https://doi.org/10.2527/2004.8261572x 15216982

[CIT0011] Gianola, D. 1982. Theory and analysis of threshold characters. J. Anim. Sci. 54:1079–1096. doi: https://doi.org/10.2527/jas1982.5451079x

[CIT0012] Hardie, L. C., B. J. Heins, and C. D. Dechow. 2021. Genetic parameters for stayability of Holsteins in US organic herds. J. Dairy Sci. 104:4507–4515. doi: https://doi.org/10.3168/jds.2020-19399 33589261

[CIT0013] Hu, H., T. Mu, Y. Ma, X. Wang, and Y. Ma. 2021. Analysis of longevity traits in Holstein cattle: a review. Front. Genet. 12:695543. doi: https://doi.org/10.3389/fgene.2021.695543 34413878 PMC8369829

[CIT0014] Hu, H. H., F. Li, T. Mu, L. Y. Han, X. F. Feng, Y. F. Ma, Y. Jiang, X. S. Xue, B. Q. Du, and R. R. Li. 2023. Genetic analysis of longevity and their associations with fertility traits in Holstein cattle. Animal. 17:100851. doi: https://doi.org/10.1016/j.animal.2023.100851 37263130

[CIT0015] Hudson, G. F. S., and L. D. Van Vleck. 1981. Relationship between production and stayability in Holstein cattle. J. Dairy Sci. 64:2246–2250. doi: https://doi.org/10.3168/jds.s0022-0302(81)82836-6

[CIT0016] Imbayarwo-Chikosi, V. E., V. Ducrocq, C. B. Banga, T. E. Halimani, J. B. Van Wyk, A. Maiwashe, and K. Dzama. 2018. Impact of conformation traits on functional longevity in South African Holstein cattle. Anim. Prod. Sci. 58:481. doi: https://doi.org/10.1071/an16387 28295685

[CIT0017] Jamrozik, J., S. McGrath, R. A. Kemp, and S. P. Miller. 2013. Estimates of genetic parameters for stayability to consecutive calvings of Canadian Simmentals by random regression models1. J. Anim. Sci. 91:3634–3643. doi: https://doi.org/10.2527/jas.2012-6126 23881688

[CIT0018] Legarra, A., O. F. Christensen, I. Aguilar, and I. Misztal. 2014. Single step, a general approach for genomic selection. Livest. Sci. 166:54–65. doi: https://doi.org/10.1016/j.livsci.2014.04.029

[CIT0019] Martinez, G. E., R. M. Koch, L. V. Cundiff, K. E. Gregory, S. D. Kachman, and L. D. Van Vleck. 2005. Genetic parameters for stayability, stayability at calving, and stayability at weaning to specified ages for Hereford cows1. J. Anim. Sci. 83:2033–2042. doi: https://doi.org/10.2527/2005.8392033x 16100057

[CIT0020] Newman, S., C. A. Morris, R. L. Baker, and G. B. Nicoll. 1992. Genetic improvement of beef cattle in New Zealand: breeding objectives. Livest. Prod. Sci. 32:111–130. doi: https://doi.org/10.1016/s0301-6226(12)80031-5

[CIT0021] Plummer, M., N. Best, K. Cowles, K. Vines, D. Sarkar, D. Bates, R. Almond, and A. Magnusson. 2006. CODA: convergence diagnosis and output analysis for MCMC 6. R News. 6:7–11. https://journal.r-project.org/articles/RN-2006-002/RN-2006-002.pdf

[CIT0022] Pollak, E. J., J. V. D. Werf, and R. L. Quaas. 1984. Selection bias and multiple trait evaluation. J. Dairy Sci. 67:1590–1595. doi: https://doi.org/10.3168/jds.S0022-0302(84)81481-2

[CIT0023] Rizzo, E. C. A., F. R. A. Neto, I. D. P. S. Diaz, M. M. Dias, R. B. Costa, H. T. Ventura, H. N. Oliveira, and A. J. S. Falcão. 2015. Genetic association of productive and reproductive traits with stayability in Nellore cattle: analysis using Bayesian models. Genet. Mol. Res. 14:14956–14966. doi: https://doi.org/10.4238/2015.November.24.3 26634457

[CIT0024] Rosen, B. D., D. M. Bickhart, R. D. Schnabel, S. Koren, C. G. Elsik, E. Tseng, T. N. Rowan, W. Y. Low, A. Zimin, C. Couldrey, et al. 2020. De novo assembly of the cattle reference genome with single-molecule sequencing. GigaScience. 9:3. doi: https://doi.org/10.1093/gigascience/giaa021 PMC7081964 32191811

[CIT0025] Santana, M. L., J. P. Eler, J. B. S. Ferraz, and E. C. Mattos. 2012. Genetic relationship between growth and reproductive traits in Nellore cattle. Animal. 6:565–570. doi: https://doi.org/10.1017/S1751731111001856 22436271

[CIT0026] Santana, M. L., J. P. Eler, A. B. Bignardi, and J. B. S. Ferraz. 2013. Genetic associations among average annual productivity, growth traits, and stayability: a parallel between Nelore and composite beef cattle1. J. Anim. Sci. 91:2566–2574. doi: https://doi.org/10.2527/jas.2012-5856 23482586

[CIT0027] Sargolzaei, M., J. P. Chesnais, and F. S. Schenkel. 2014. A new approach for efficient genotype imputation using information from relatives. BMC Genomics. 15:478. doi: https://doi.org/10.1186/1471-2164-15-478 24935670 PMC4076979

[CIT0028] Sewalem, A., G. J. Kistemaker, F. Miglior, and B. J. Van Doormaal. 2004. Analysis of the relationship between type traits and functional survival in Canadian Holsteins using a Weibull proportional hazards model. J. Dairy Sci. 87:3938–3946. doi: https://doi.org/10.3168/jds.S0022-0302(04)73533-X 15483178

[CIT0029] Sewalem, A., G. J. Kistemaker, V. Ducrocq, and B. J. V. Doormaal. 2005. Genetic analysis of herd life in Canadian dairy cattle on a lactation basis using a Weibull proportional hazards model. J. Dairy Sci. 88:368–375. doi: https://doi.org/10.3168/jds.S0022-0302(05)72696-5 15591401

[CIT0030] Silva, D. O., M. L. Santana, D. R. Ayres, G. R. O. Menezes, L. O. C. Silva, P. R. C. Nobre, and R. J. Pereira. 2018. Genetic parameters for stayability to consecutive calvings in Zebu cattle. Animal. 12:1807–1814. doi: https://doi.org/10.1017/S1751731117003457 29268814

[CIT0031] Silva, D. O., G. A. Fernandes Júnior, L. F. S. Fonseca, L. F. M. Mota, T. Bresolin, R. Carvalheiro, and L. G. De Albuquerque. 2024. Genome-wide association study for stayability at different calvings in Nellore beef cattle. BMC Genomics. 25:93. doi: https://doi.org/10.1186/s12864-024-10020-y 38254039 PMC10804543

[CIT0032] Smith, S. P. 1990. Survival, endurance and censored observations in animal breeding. In: Gianola, D., K. Hammond, editors. Advances in statistical methods for genetic improvement of livestock. Berlin, Heidelberg: Springer Berlin Heidelberg; p. 344–360. doi: https://doi.org/10.1007/978-3-642-74487-7_16

[CIT0033] Stephen, M. A., C. R. Burke, J. E. Pryce, N. M. Steele, P. R. Amer, S. Meier, C. V. C. Clair, and D. J. Garrick. 2023. Comparison of methods for deriving phenotypes from incomplete observation data with an application to age at puberty in dairy cattle. J. Anim. Sci. Biotechnol. 14:119. doi: https://doi.org/10.1186/s40104-023-00921-5 37684681 PMC10492402

[CIT0034] Van Melis, M. H., J. P. Eler, H. N. Oliveir, G. J. M. Rosa, J. A. V. Silva, J. B. S. Ferraz, and E. Pereira. 2007. Study of stayability in Nellore cows using a threshold model1. J. Anim. Sci. 85:1780–1786. doi: https://doi.org/10.2527/jas.2005-608 17371792

[CIT0035] VanRaden, P. M. 2008. Efficient methods to compute genomic predictions. J. Dairy Sci. 91:4414–4423. doi: https://doi.org/10.3168/jds.2007-0980 18946147

[CIT0036] Varona, L., C. Moreno, and J. Altarriba. 2012. Genetic correlation of longevity with growth, post-mortem, docility and some morphological traits in the Pirenaica beef cattle breed. Animal. 6:873–879. doi: https://doi.org/10.1017/S1751731111002072 22558956

