# Supplementary material for: Genetic parameters of functional longevity and associated traits in Italian Charolais and Limousine breeds
Source: J Anim Sci. 2024 Nov 18;102:skae354. doi: 10.1093/jas/skae354 (PMC11630839; doi:10.1093/jas/skae354)
Supplement: skae354_suppl_Supplementary_Tables_S1-S4_Figures_S1-S2 [file skae354_suppl_supplementary_tables_s1-s4_figures_s1-s2.docx]

**Supplementary Table 1.** Total number of cows used in the analyses and the percentage of censored data for each stayability (STAY) record, categorized by the number of records for Limousine and Charolais breeds.

| Trait | Definition | Limousine | | Charolais | |
| --- | --- | --- | --- | --- | --- |
|  |  | N^2^ | Censored (%) | N^2^ | Censored (%) |
| STAY1 | Stayability as a first parity = 1; failed = 0 | 38,188 | 0 | 9,174 | 0 |
| STAY2 | Stayability as a second parity = 1; failed = 0 | 33,209 | 13.04 | 8,071 | 12.02 |
| STAY3 | Stayability as a third parity = 1; failed = 0 | 30,105 | 21.17 | 7,519 | 18.04 |
| STAY4 | Stayability as a fourth parity = 1; failed = 0 | 27,978 | 26.74 | 7,078 | 22.85 |
| STAY5 | Stayability as a fifth parity = 1; failed = 0 | 26,546 | 30.49 | 6,774 | 26.16 |
| STAY6 | Stayability as a sixth parity = 1; failed = 0 | 25,372 | 33.56 | 6,550 | 28.60 |
| STAY7 | Stayability as a seventh parity = 1; failed = 0 | 24,429 | 36.03 | 6,381 | 30.45 |
| STAY8 | Stayability as a eight parity = 1; failed = 0 | 23,657 | 38.05 | 6,245 | 31.93 |

^1^ Continuity in herd until the subsequent parity (Success = 1; Failure = 0)

^2^ Total number of cows, including both those that survived and were culled (used in the genetic analyses), excluding censored data (which were treated as missing in the model)

**Supplementary Table 2.** Number of total conformation traits considered with the relative definition and related score for Limousine and Charolais.

| Trait | Definition | N^1^ | | Mean ± SD | |
| --- | --- | --- | --- | --- | --- |
|  |  | Limousine | Charolais | Limousine | Charolais |
| Wither width | It is defined by the distance between the scapulae | 32,119 | 7,537 | 5.86 ± 1.03 | 5.91 ± 0.92 |
| Rump convexity | It is observed from the side, along with the width of the rear, and gives an idea of the development of the most valuable muscles of the thigh | 32,119 | 7,537 | 6.04 ± 1.03 | 5.84 ± 1.07 |
| Rump length | It is observed from the side and gives an idea of the length of the most valuable muscles of the thigh | 32,119 | 7,537 | 5.82 ± 0.98 | 5.66 ± 1.01 |
| Dorsolumbar line length | It measures the distance between the withers and the tip of the hips; in practice, it is measured by observing the distance between the front and rear legs, taking into account the development of the animal | 32,119 | 7,537 | 6.51 ± 0.88 | 6.54 ± 0.84 |
| Rear width | It is defined by the width of the thigh muscle, evaluated at mid-height | 32,119 | 7,537 | 6.09 ± 1.04 | 6.10 ± 1.01 |
| Back width | It defines the importance of the muscle just behind the scapulae | 32,119 | 7,537 | 5.59 ± 1.12 | 5.50 ± 1.11 |
| Pelvic length | It defines the length between the hips and the ischium and must be proportionate to the loin length | 32,119 | 7,537 | 5.99 ± 0.89 | 6.01 ± 0.90 |
| Development | It is defined by the size of the animal, which can be appreciated based on the height at the withers | 32,119 | 7,537 | 6.05 ± 1.01 | 6.10 ± 0.93 |

^1^ Total number of cows per each of the conformation traits considered from STAY2

**Supplementary Table 3.** Estimates of heritability, intra-herd heritability, and herd effect for stay-ability (STAY) traits for Limousine and Charolais using a single step GBLUP approach considering stayability (Gaussian-linear model) and liability of stayability (threshold model). Values within parentheses represent Highest Posterior Density Interval (HPDI).

| Trait^1^ | Limousine | | | | | |  | Charolais | | | | | |
| --- | --- | --- | --- | --- | --- | --- | --- | --- | --- | --- | --- | --- | --- |
|  | Gaussian-linear model | | | Threshold model | | |  | Gaussian-linear model | | | Threshold model | | |
|  | h^2^ | $h_{\mathrm{IH}}^{2}$ | $h_{i}$ | h^2^ | $h_{\mathrm{IH}}^{2}$ | $h_{i}$ |  | h^2^ | $h_{\mathrm{IH}}^{2}$ | $h_{i}$ | h^2^ | $h_{\mathrm{IH}}^{2}$ | $h_{i}$ |
| STAY2 | 0.13 ^(0.11; 0.16)^ | 0.18 ^(0.15; 0.21)^ | 0.25 ^(0.23; 0.27)^ | 0.21 ^(0.17; 0.24)^ | 0.32 ^(0.26; 0.38)^ | 0.34 ^(0.31; 0.37)^ |  | 0.14 ^(0.10; 0.18)^ | 0.17 ^(0.11; 0.22)^ | 0.20 ^(0.16; 0.23)^ | 0.21 ^(0.14; 0.27)^ | 0.30 ^(0.20; 0.40)^ | 0.30 ^(0.25; 0.36)^ |
| STAY3 | 0.12 ^(0.10; 0.14)^ | 0.16 ^(0.13; 0.19)^ | 0.24 ^(0.22; 0.26)^ | 0.18 ^(0.15; 0.21)^ | 0.30 ^(0.25; 0.35)^ | 0.39 ^(0.36; 0.42)^ |  | 0.08 ^(0.04; 0.13)^ | 0.11 ^(0.06; 0.15)^ | 0.23 ^(0.19; 0.26)^ | 0.11 ^(0.06; 0.17)^ | 0.19 ^(0.11; 0.26)^ | 0.40 ^(0.34; 0.44)^ |
| STAY4 | 0.13 ^(0.10; 0.15)^ | 0.17 ^(0.13; 0.19)^ | 0.22 ^(0.20; 0.24)^ | 0.19 ^(0.16; 0.22)^ | 0.34 ^(0.29; 0.38)^ | 0.43 ^(0.40; 0.46)^ |  | 0.09 ^(0.04; 0.11)^ | 0.11 ^(0.06; 0.15)^ | 0.20 ^(0.17; 0.24)^ | 0.11 ^(0.05; 0.15)^ | 0.20 ^(0.13; 0.28)^ | 0.40 ^(0.34; 0.45)^ |
| STAY5 | 0.13 ^(0.11; 0.15)^ | 0.16 ^(0.13; 0.18)^ | 0.20 ^(0.18; 0.21)^ | 0.18 ^(0.15; 0.21)^ | 0.33 ^(0.26; 0.39)^ | 0.45 ^(0.41; 0.48)^ |  | 0.11 ^(0.06; 0.13)^ | 0.13 ^(0.09; 0.18)^ | 0.20 ^(0.17; 0.24)^ | 0.16 ^(0.10; 0.21)^ | 0.27 ^(0.18; 0.36)^ | 0.43 ^(0.36; 0.49)^ |
| STAY6 | 0.11 ^(0.09; 0.13)^ | 0.13 ^(0.11; 0.15)^ | 0.17 ^(0.15; 0.19)^ | 0.16 ^(0.12; 0.19)^ | 0.30 ^(0.26; 0.35)^ | 0.45 ^(0.41; 0.49)^ |  | 0.11 ^(0.07; 0.15)^ | 0.14 ^(0.09; 0.18)^ | 0.17 ^(0.15; 0.21)^ | 0.17 ^(0.11; 0.23)^ | 0.31 ^(0.22; 0.41)^ | 0.45 ^(0.38; 0.53)^ |
| STAY7 | 0.10 ^(0.08; 0.12)^ | 0.12 ^(0.10; 0.14)^ | 0.16 ^(0.14; 0.17)^ | 0.10 ^(0.08; 0.13)^ | 0.23 ^(0.19; 0.28)^ | 0.51 ^(0.45; 0.56)^ |  | 0.13 ^(0.08; 0.16)^ | 0.15 ^(0.11; 0.19)^ | 0.14 ^(0.11; 0.17)^ | 0.24 ^(0.17; 0.31)^ | 0.40 ^(0.30; 0.50)^ | 0.41 ^(0.33; 0.49)^ |
| STAY8 | 0.11 ^(0.10; 0.13)^ | 0.13 ^(0.11; 0.15)^ | 0.15 ^(0.13; 0.16)^ | 0.14 ^(0.02; 0.20)^ | 0.33 ^(0.26; 0.41)^ | 0.56 ^(0.43; 0.91)^ |  | 0.11 ^(0.07; 0.14)^ | 0.12 ^(0.08; 0.16)^ | 0.12 ^(0.10; 0.15)^ | 0.19 ^(0.10; 0.26)^ | 0.34 ^(0.18; 0.44)^ | 0.45 ^(0.36; 0.54)^ |

Abbreviations: h^2^ = heritability; $h_{\mathrm{IH}}^{2}=$intra-herd heritability; $h_{i}$= herd effect.

^1^STAY2 = stayability to parity 2; STAY3 = stayability to parity 3; STAY4 = stayability to parity 4; STAY5 = stayability to parity 5; STAY6 = stayability to parity 6; STAY7 = stayability to parity 7; STAY8 = stayability to parity 8.

**Supplementary table 4.** Estimates of variance components for STAY traits Limousine and Charolais using a single step GBLUP approach considering stayability (Gaussian-linear model) and liability of stayability (threshold model). Values within parentheses represent Highest Posterior Density Interval (HPDI).

| Trait^1^ | Limousine | | | |  | Charolais | | | |
| --- | --- | --- | --- | --- | --- | --- | --- | --- | --- |
|  | Gaussian-linear model | | Threshold model | |  | Gaussian-linear model | | Threshold model | |
|  | $\sigma_{a}^{2}$ | $\sigma_{h}^{2}$ | $\sigma_{a}^{2}$ | $\sigma_{h}^{2}$ |  | $\sigma_{a}^{2}$ | $\sigma_{h}^{2}$ | $\sigma_{a}^{2}$ | $\sigma_{h}^{2}$ |
| STAY2 | 16.21 ^(13.40; 19.39)^ | 30.20 ^(27.07; 32.96)^ | 0.46 ^(0.35; 0.56)^ | 0.76 ^(0.66; 0.86)^ |  | 19.56 ^(14.19; 25.11)^ | 28.06 ^(22.260 34.21)^ | 0.43 ^(0.23; 0.61)^ | 0.63 ^(0.48; 0.81)^ |
| STAY3 | 17.69 ^(14.33; 21.13)^ | 35.42 ^(31.88; 38.97)^ | 0.43 ^(0.35; 0.52)^ | 0.93 ^(0.80; 1.04)^ |  | 13.05 ^(6.91; 19.89)^ | 35.63 ^(29.30; 42.88)^ | 0.24 ^(0.11; 0.38)^ | 0.82 ^(0.65; 1.01)^ |
| STAY4 | 18.81 ^(15.74; 22.62)^ | 32.16 ^(28.97; 35.24)^ | 0.49 ^(0.37; 0.60)^ | 1.11 ^(0.96; 1.23)^ |  | 10.89 ^(5.92; 16.51)^ | 28.96 ^(23.80; 35.18)^ | 0.22 ^(0.09; 0.34)^ | 0.81 ^(0.63; 1.04)^ |
| STAY5 | 17.53 ^(14.59; 20.47)^ | 27.12 ^(24.47; 29.92)^ | 0.50 ^(0.39; 0.61)^ | 1.22 ^(1.05; 1.42)^ |  | 11.80 ^(7.21; 15.78)^ | 24.32 ^(19.57; 29.20)^ | 0.33 ^(0.16; 0.48)^ | 1.01 ^(0.75; 1.27)^ |
| STAY6 | 13.54 ^(11.25; 15.87)^ | 21.34 ^(18.99; 23.59)^ | 0.41 ^(0.31; 0.51)^ | 1.14 ^(0.97; 1.23)^ |  | 10.97 ^(7.23; 14.69)^ | 17.37 ^(13.95; 21.13)^ | 0.42 ^(0.22; 0.63)^ | 1.17 ^(0.85; 1.54)^ |
| STAY7 | 11.14 ^(9.24; 13.25)^ | 16.91 ^(14.99; 18.93)^ | 0.25 ^(0.19; 0.32)^ | 1.24 ^(0.99; 1.50)^ |  | 9.29 ^(6.30; 12.70)^ | 10.96 ^(8.71; 13.50)^ | 0.63 ^(0.35; 0.97)^ | 1.15 ^(0.81; 1.6)^ |
| STAY8 | 10.61 ^(8.92; 12.17)^ | 13.63 ^(12.21; 15.12)^ | 0.45 ^(0.29; 0.62)^ | 2.98 ^(0.76; 10.75)^ |  | 5.92 ^(3.78; 8.20)^ | 7.09 ^(5.48; 8.70)^ | 0.45 ^(0.24; 0.66)^ | 1.20 ^(0.78; 1.64)^ |

Abbreviations: $\sigma_{a}^{2}$ = additive genetic variance; $\sigma_{h}^{2}$= herd variance.

^1^STAY2 = stayability to parity 2; STAY3 = stayability to parity 3; STAY4 = stayability to parity 4; STAY5 = stayability to parity 5; STAY6 = stayability to parity 6; STAY7 = stayability to parity 7; STAY8 = stayability to parity 8.

**Supplementary figure 1.** Trends of herd effect with the relative SE for stay-ability (STAY) traits (STAY2 until STAY8) for Limousine and Charolais using a single step GBLUP approach considering STAY (Gaussian-linear model) and liability of STAY (threshold model), respectively.


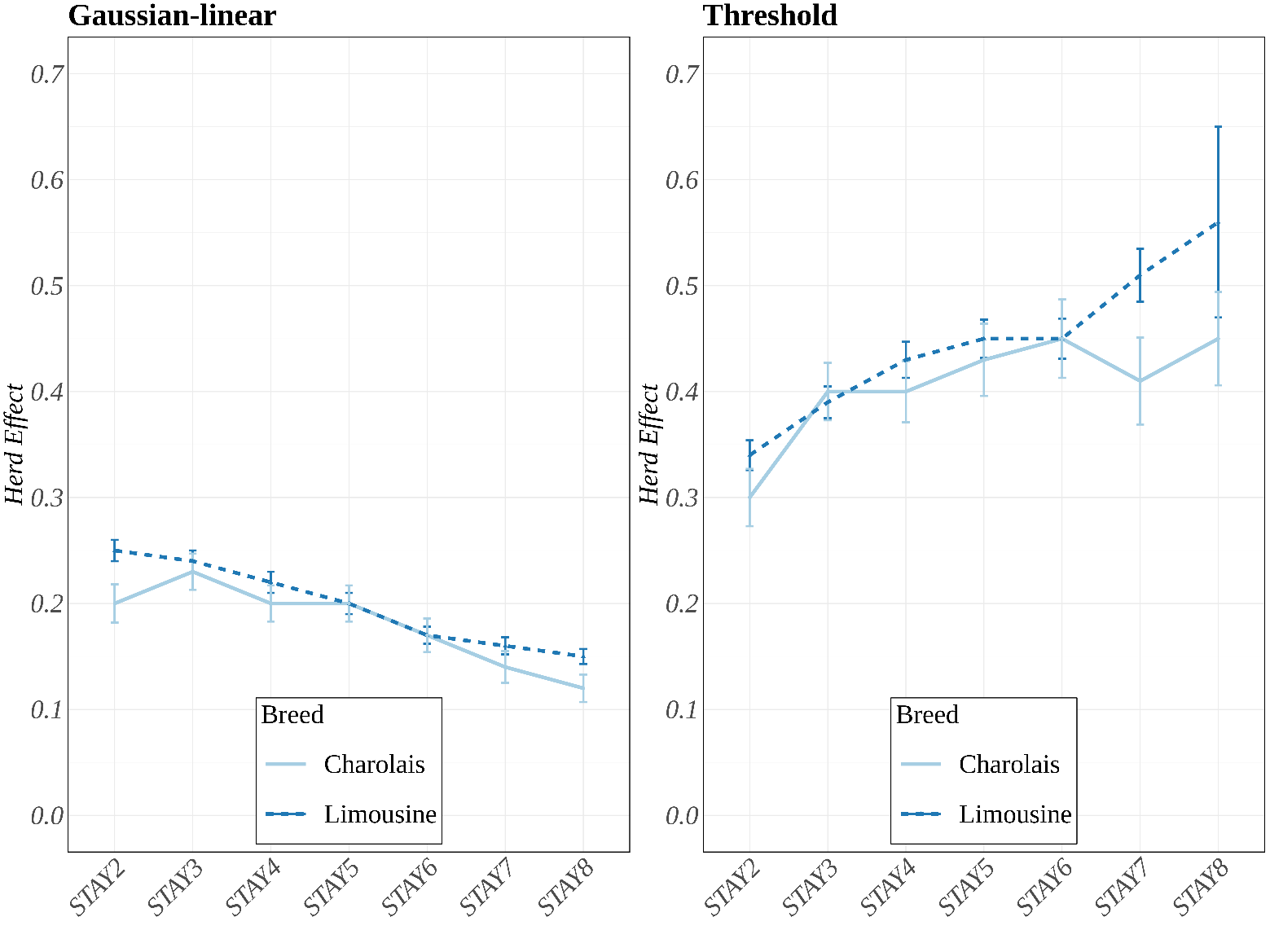


**Supplementary figure 2.** Trends of intra-herd heritability with the relative SE for stay-ability (STAY) traits (STAY2 until STAY8) for Limousine and Charolais using a single step GBLUP approach considering STAY (Gaussian-linear model) and liability of STAY (threshold model), respectively.

**
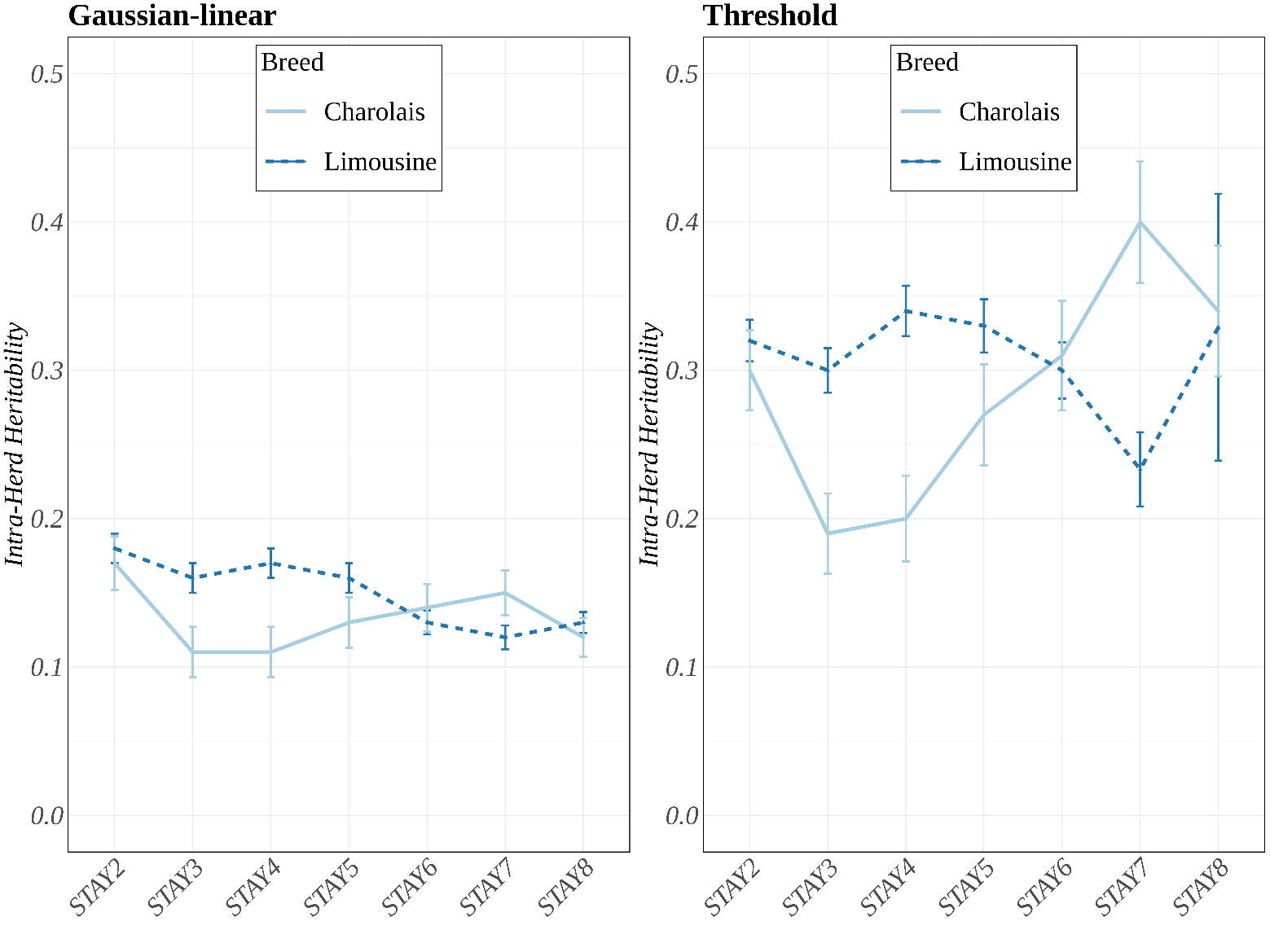
**
